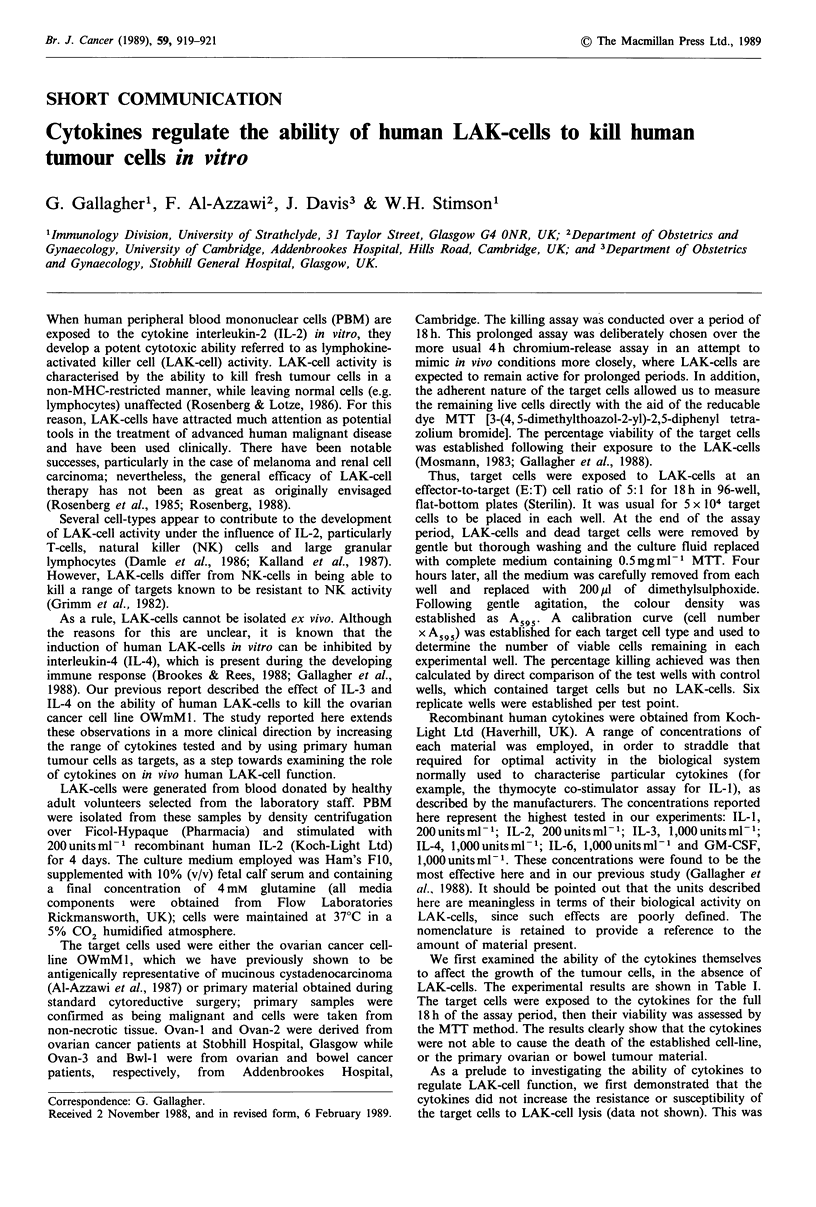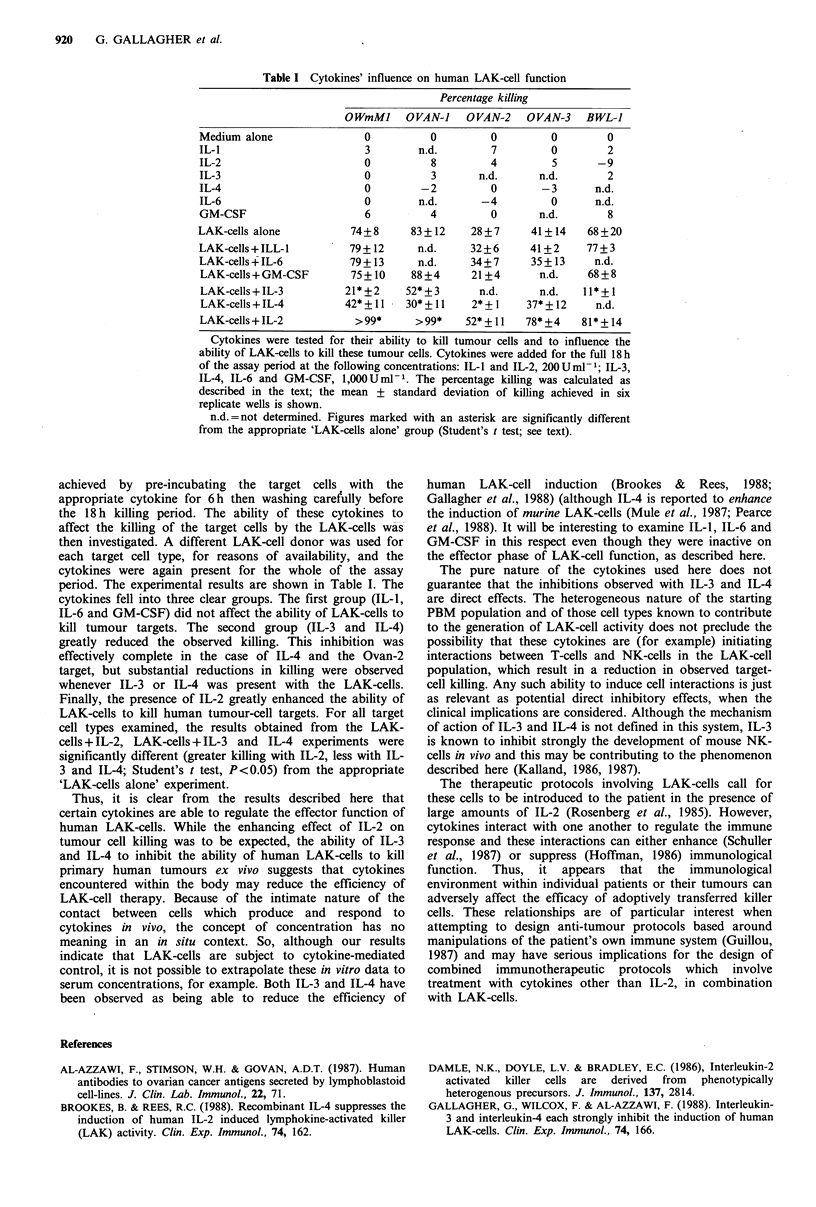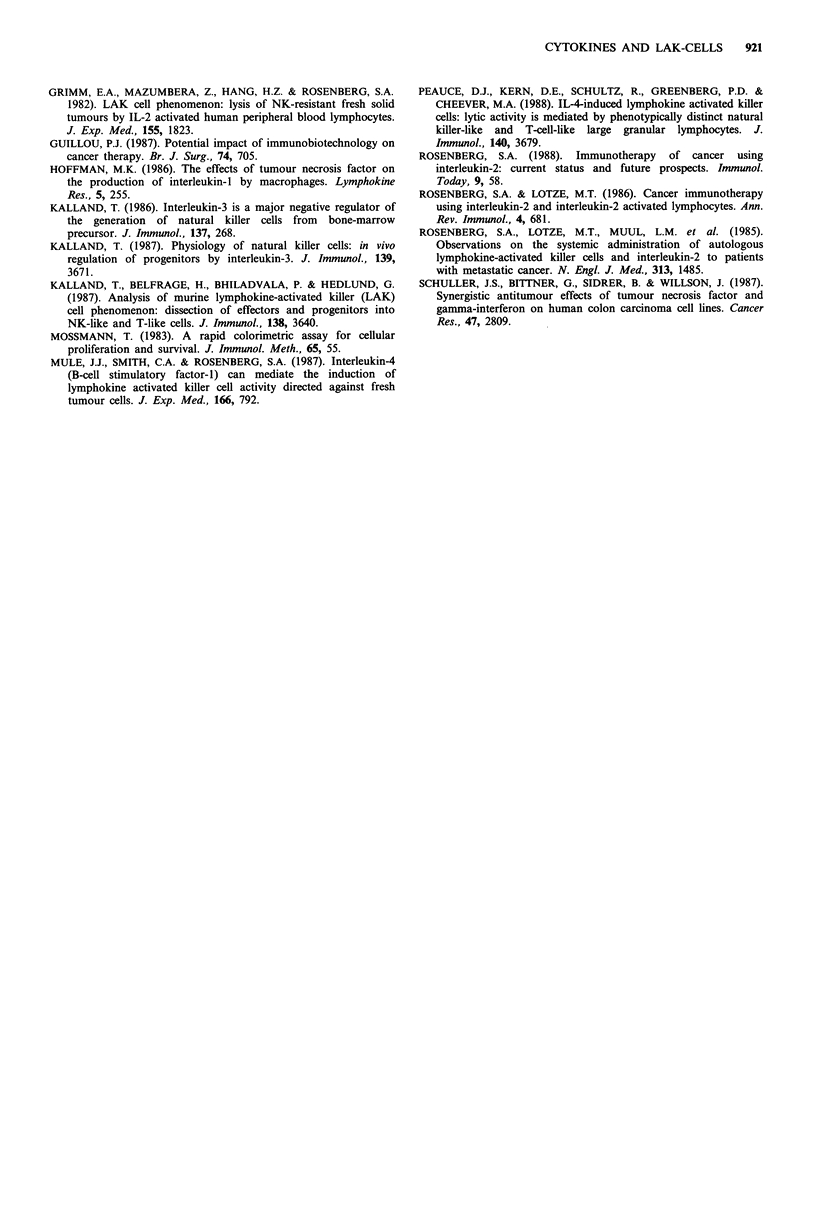# Cytokines regulate the ability of human LAK-cells to kill human tumour cells in vitro.

**DOI:** 10.1038/bjc.1989.194

**Published:** 1989-06

**Authors:** G. Gallagher, F. al-Azzawi, J. Davis, W. H. Stimson

**Affiliations:** Immunology Division, University of Strathclyde, Glasgow, UK.


Br. J. Cancer (1989), 59, 919–921 © The Macmillan Press Ltd., 1989

SHORT COMMUNICATION

### Cytokines regulate the ability of human LAK-cells to kill human tumour cells *in vitro*

G. Gallagher[1], F. Al-Azzawi[2], J. Davis[3] & W.H. Stimson[1]

[1]*Immunology Division, University of Strathclyde, 31 Taylor Street, Glasgow G4 0NR, UK;* [2]*Department of Obstetrics and Gynaecology, University of Cambridge, Addenbrookes Hospital, Hills Road, Cambridge, UK; and* [3]*Department of Obstetrics and Gynaecology, Stobhill General Hospital, Glasgow, UK.*

When human peripheral blood mononuclear cells (PBM) are exposed to the cytokine interleukin-2 (IL-2) *in vitro*, they develop a potent cytotoxic ability referred to as lymphokine-activated killer cell (LAK-cell) activity. LAK-cell activity is characterised by the ability to kill fresh tumour cells in a non-MHC-restricted manner, while leaving normal cells (e.g. lymphocytes) unaffected (Rosenberg & Lotze, 1986). For this reason, LAK-cells have attracted much attention as potential tools in the treatment of advanced human malignant disease and have been used clinically. There have been notable successes, particularly in the case of melanoma and renal cell carcinoma; nevertheless, the general efficacy of LAK-cell therapy has not been as great as originally envisaged (Rosenberg *et al.*, 1985; Rosenberg, 1988).

Several cell-types appear to contribute to the development of LAK-cell activity under the influence of IL-2, particularly T-cells, natural killer (NK) cells and large granular lymphocytes (Damle *et al.*, 1986; Kalland *et al.*, 1987). However, LAK-cells differ from NK-cells in being able to kill a range of targets known to be resistant to NK activity (Grimm *et al.*, 1982).

As a rule, LAK-cells cannot be isolated *ex vivo*. Although the reasons for this are unclear, it is known that the induction of human LAK-cells *in vitro* can be inhibited by interleukin-4 (IL-4), which is present during the developing immune response (Brookes & Rees, 1988; Gallagher *et al.*, 1988). Our previous report described the effect of IL-3 and IL-4 on the ability of human LAK-cells to kill the ovarian cancer cell line OWmM1. The study reported here extends these observations in a more clinical direction by increasing the range of cytokines tested and by using primary human tumour cells as targets, as a step towards examining the role of cytokines on *in vivo* human LAK-cell function.

LAK-cells were generated from blood donated by healthy adult volunteers selected from the laboratory staff. PBM were isolated from these samples by density centrifugation over Ficol-Hypaque (Pharmacia) and stimulated with 200 units ml$^{-1}$ recombinant human IL-2 (Koch-Light Ltd) for 4 days. The culture medium employed was Ham's F10, supplemented with 10% (v/v) fetal calf serum and containing a final concentration of 4 mM glutamine (all media components were obtained from Flow Laboratories Rickmansworth, UK); cells were maintained at 37°C in a 5% $CO_2$ humidified atmosphere.

The target cells used were either the ovarian cancer cell-line OWmM1, which we have previously shown to be antigenically representative of mucinous cystadenocarcinoma (Al-Azzawi *et al.*, 1987) or primary material obtained during standard cytoreductive surgery; primary samples were confirmed as being malignant and cells were taken from non-necrotic tissue. Ovan-1 and Ovan-2 were derived from ovarian cancer patients at Stobhill Hospital, Glasgow while Ovan-3 and Bwl-1 were from ovarian and bowel cancer patients, respectively, from Addenbrookes Hospital, Cambridge. The killing assay was conducted over a period of 18 h. This prolonged assay was deliberately chosen over the more usual 4 h chromium-release assay in an attempt to mimic *in vivo* conditions more closely, where LAK-cells are expected to remain active for prolonged periods. In addition, the adherent nature of the target cells allowed us to measure the remaining live cells directly with the aid of the reducable dye MTT [3-(4,5-dimethylthoazol-2-yl)-2,5-diphenyl tetrazolium bromide]. The percentage viability of the target cells was established following their exposure to the LAK-cells (Mosmann, 1983; Gallagher *et al.*, 1988).

Correspondence: G. Gallagher.
Received 2 November 1988, and in revised form, 6 February 1989.

Thus, target cells were exposed to LAK-cells at an effector-to-target (E:T) cell ratio of 5:1 for 18 h in 96-well, flat-bottom plates (Sterilin). It was usual for $5 \times 10^4$ target cells to be placed in each well. At the end of the assay period, LAK-cells and dead target cells were removed by gentle but thorough washing and the culture fluid replaced with complete medium containing 0.5 mg ml$^{-1}$ MTT. Four hours later, all the medium was carefully removed from each well and replaced with 200 µl of dimethylsulphoxide. Following gentle agitation, the colour density was established as $A_{595}$. A calibration curve (cell number $\times A_{595}$) was established for each target cell type and used to determine the number of viable cells remaining in each experimental well. The percentage killing achieved was then calculated by direct comparison of the test wells with control wells, which contained target cells but no LAK-cells. Six replicate wells were established per test point.

Recombinant human cytokines were obtained from Koch-Light Ltd (Haverhill, UK). A range of concentrations of each material was employed, in order to straddle that required for optimal activity in the biological system normally used to characterise particular cytokines (for example, the thymocyte co-stimulator assay for IL-1), as described by the manufacturers. The concentrations reported here represent the highest tested in our experiments: IL-1, 200 units ml$^{-1}$; IL-2, 200 units ml$^{-1}$; IL-3, 1,000 units ml$^{-1}$; IL-4, 1,000 units ml$^{-1}$; IL-6, 1,000 units ml$^{-1}$ and GM-CSF, 1,000 units ml$^{-1}$. These concentrations were found to be the most effective here and in our previous study (Gallagher *et al.*, 1988). It should be pointed out that the units described here are meaningless in terms of their biological activity on LAK-cells, since such effects are poorly defined. The nomenclature is retained to provide a reference to the amount of material present.

We first examined the ability of the cytokines themselves to affect the growth of the tumour cells, in the absence of LAK-cells. The experimental results are shown in Table I. The target cells were exposed to the cytokines for the full 18 h of the assay period, then their viability was assessed by the MTT method. The results clearly show that the cytokines were not able to cause the death of the established cell-line, or the primary ovarian or bowel tumour material.

As a prelude to investigating the ability of cytokines to regulate LAK-cell function, we first demonstrated that the cytokines did not increase the resistance or susceptibility of the target cells to LAK-cell lysis (data not shown). This was

**Table I** Cytokines' influence on human LAK-cell function

| | *Percentage killing* | | | | |
|---|---|---|---|---|---|
| | *OWmM1* | *OVAN-1* | *OVAN-2* | *OVAN-3* | *BWL-1* |
| Medium alone | 0 | 0 | 0 | 0 | 0 |
| IL-1 | 3 | n.d. | 7 | 0 | 2 |
| IL-2 | 0 | 8 | 4 | 5 | −9 |
| IL-3 | 0 | 3 | n.d. | n.d. | 2 |
| IL-4 | 0 | −2 | 0 | −3 | n.d. |
| IL-6 | 0 | n.d. | −4 | 0 | n.d. |
| GM-CSF | 6 | 4 | 0 | n.d. | 8 |
| LAK-cells alone | 74±8 | 83±12 | 28±7 | 41±14 | 68±20 |
| LAK-cells+ILL-1 | 79±12 | n.d. | 32±6 | 41±2 | 77±3 |
| LAK-cells+IL-6 | 79±13 | n.d. | 34±7 | 35±13 | n.d. |
| LAK-cells+GM-CSF | 75±10 | 88±4 | 21±4 | n.d. | 68±8 |
| LAK-cells+IL-3 | 21*±2 | 52*±3 | n.d. | n.d. | 11*±1 |
| LAK-cells+IL-4 | 42*±11 | 30*±11 | 2*±1 | 37*±12 | n.d. |
| LAK-cells+IL-2 | >99* | >99* | 52*±11 | 78*±4 | 81*±14 |

Cytokines were tested for their ability to kill tumour cells and to influence the ability of LAK-cells to kill these tumour cells. Cytokines were added for the full 18 h of the assay period at the following concentrations: IL-1 and IL-2, 200 U ml$^{-1}$; IL-3, IL-4, IL-6 and GM-CSF, 1,000 U ml$^{-1}$. The percentage killing was calculated as described in the text; the mean ± standard deviation of killing achieved in six replicate wells is shown.

n.d. = not determined. Figures marked with an asterisk are significantly different from the appropriate 'LAK-cells alone' group (Student's *t* test; see text).

achieved by pre-incubating the target cells with the appropriate cytokine for 6 h then washing carefully before the 18 h killing period. The ability of these cytokines to affect the killing of the target cells by the LAK-cells was then investigated. A different LAK-cell donor was used for each target cell type, for reasons of availability, and the cytokines were again present for the whole of the assay period. The experimental results are shown in Table I. The cytokines fell into three clear groups. The first group (IL-1, IL-6 and GM-CSF) did not affect the ability of LAK-cells to kill tumour targets. The second group (IL-3 and IL-4) greatly reduced the observed killing. This inhibition was effectively complete in the case of IL-4 and the Ovan-2 target, but substantial reductions in killing were observed whenever IL-3 or IL-4 was present with the LAK-cells. Finally, the presence of IL-2 greatly enhanced the ability of LAK-cells to kill human tumour-cell targets. For all target cell types examined, the results obtained from the LAK-cells+IL-2, LAK-cells+IL-3 and IL-4 experiments were significantly different (greater killing with IL-2, less with IL-3 and IL-4; Student's *t* test, $P<0.05$) from the appropriate 'LAK-cells alone' experiment.

Thus, it is clear from the results described here that certain cytokines are able to regulate the effector function of human LAK-cells. While the enhancing effect of IL-2 on tumour cell killing was to be expected, the ability of IL-3 and IL-4 to inhibit the ability of human LAK-cells to kill primary human tumours *ex vivo* suggests that cytokines encountered within the body may reduce the efficiency of LAK-cell therapy. Because of the intimate nature of the contact between cells which produce and respond to cytokines *in vivo*, the concept of concentration has no meaning in an *in situ* context. So, although our results indicate that LAK-cells are subject to cytokine-mediated control, it is not possible to extrapolate these *in vitro* data to serum concentrations, for example. Both IL-3 and IL-4 have been observed as being able to reduce the efficiency of human LAK-cell induction (Brookes & Rees, 1988; Gallagher *et al.*, 1988) (although IL-4 is reported to *enhance* the induction of *murine* LAK-cells (Mule *et al.*, 1987; Pearce *et al.*, 1988). It will be interesting to examine IL-1, IL-6 and GM-CSF in this respect even though they were inactive on the effector phase of LAK-cell function, as described here.

The pure nature of the cytokines used here does not guarantee that the inhibitions observed with IL-3 and IL-4 are direct effects. The heterogeneous nature of the starting PBM population and of those cell types known to contribute to the generation of LAK-cell activity does not preclude the possibility that these cytokines are (for example) initiating interactions between T-cells and NK-cells in the LAK-cell population, which result in a reduction in observed target-cell killing. Any such ability to induce cell interactions is just as relevant as potential direct inhibitory effects, when the clinical implications are considered. Although the mechanism of action of IL-3 and IL-4 is not defined in this system, IL-3 is known to inhibit strongly the development of mouse NK-cells *in vivo* and this may be contributing to the phenomenon described here (Kalland, 1986, 1987).

The therapeutic protocols involving LAK-cells call for these cells to be introduced to the patient in the presence of large amounts of IL-2 (Rosenberg *et al.*, 1985). However, cytokines interact with one another to regulate the immune response and these interactions can either enhance (Schuller *et al.*, 1987) or suppress (Hoffman, 1986) immunological function. Thus, it appears that the immunological environment within individual patients or their tumours can adversely affect the efficacy of adoptively transferred killer cells. These relationships are of particular interest when attempting to design anti-tumour protocols based around manipulations of the patient's own immune system (Guillou, 1987) and may have serious implications for the design of combined immunotherapeutic protocols which involve treatment with cytokines other than IL-2, in combination with LAK-cells.

### References

AL-AZZAWI, F., STIMSON, W.H. & GOVAN, A.D.T. (1987). Human antibodies to ovarian cancer antigens secreted by lymphoblastoid cell-lines. *J. Clin. Lab. Immunol.*, **22**, 71.

BROOKES, B. & REES, R.C. (1988). Recombinant IL-4 suppresses the induction of human IL-2 induced lymphokine-activated killer (LAK) activity. *Clin. Exp. Immunol.*, **74**, 162.

DAMLE, N.K., DOYLE, L.V. & BRADLEY, E.C. (1986), Interleukin-2 activated killer cells are derived from phenotypically heterogenous precursors. *J. Immunol.*, **137**, 2814.

GALLAGHER, G., WILCOX, F. & AL-AZZAWI, F. (1988). Interleukin-3 and interleukin-4 each strongly inhibit the induction of human LAK-cells. *Clin. Exp. Immunol.*, **74**, 166.

GRIMM, E.A., MAZUMBERA, Z., HANG, H.Z. & ROSENBERG, S.A. 1982). LAK cell phenomenon: lysis of NK-resistant fresh solid tumours by IL-2 activated human peripheral blood lymphocytes. *J. Exp. Med.*, **155**, 1823.

GUILLOU, P.J. (1987). Potential impact of immunobiotechnology on cancer therapy. *Br. J. Surg.*, **74**, 705.

HOFFMAN, M.K. (1986). The effects of tumour necrosis factor on the production of interleukin-1 by macrophages. *Lymphokine Res.*, **5**, 255.

KALLAND, T. (1986). Interleukin-3 is a major negative regulator of the generation of natural killer cells from bone-marrow precursor. *J. Immunol.*, **137**, 268.

KALLAND, T. (1987). Physiology of natural killer cells: *in vivo* regulation of progenitors by interleukin-3. *J. Immunol.*, **139**, 3671.

KALLAND, T., BELFRAGE, H., BHILADVALA, P. & HEDLUND, G. (1987). Analysis of murine lymphokine-activated killer (LAK) cell phenomenon: dissection of effectors and progenitors into NK-like and T-like cells. *J. Immunol.*, **138**, 3640.

MOSSMANN, T. (1983). A rapid colorimetric assay for cellular proliferation and survival. *J. Immunol. Meth.*, **65**, 55.

MULE, J.J., SMITH, C.A. & ROSENBERG, S.A. (1987). Interleukin-4 (B-cell stimulatory factor-1) can mediate the induction of lymphokine activated killer cell activity directed against fresh tumour cells. *J. Exp. Med.*, **166**, 792.

PEAUCE, D.J., KERN, D.E., SCHULTZ, R., GREENBERG, P.D. & CHEEVER, M.A. (1988). IL-4-induced lymphokine activated killer cells: lytic activity is mediated by phenotypically distinct natural killer-like and T-cell-like large granular lymphocytes. *J. Immunol.*, **140**, 3679.

ROSENBERG, S.A. (1988). Immunotherapy of cancer using interleukin-2: current status and future prospects. *Immunol. Today*, **9**, 58.

ROSENBERG, S.A. & LOTZE, M.T. (1986). Cancer immunotherapy using interleukin-2 and interleukin-2 activated lymphocytes. *Ann. Rev. Immunol.*, **4**, 681.

ROSENBERG, S.A., LOTZE, M.T., MUUL, L.M. *et al.* (1985). Observations on the systemic administration of autologous lymphokine-activated killer cells and interleukin-2 to patients with metastatic cancer. *N. Engl. J. Med.*, **313**, 1485.

SCHULLER, J.S., BITTNER, G., SIDRER, B. & WILLSON, J. (1987). Synergistic antitumour effects of tumour necrosis factor and gamma-interferon on human colon carcinoma cell lines. *Cancer Res.*, **47**, 2809.